# Reply: The role of *DNAJB2* in amyotrophic lateral sclerosis

**DOI:** 10.1093/brain/aww155

**Published:** 2016-06-21

**Authors:** Han-Jou Chen, Christopher E. Shaw

**Affiliations:** Maurice Wohl Clinical Neuroscience Institute, Institute of Psychiatry, Psychology and Neuroscience, King’s College London, London, UK

Sir,

Heat shock proteins (HSPs) are protein chaperones that play important roles in maintaining protein homeostasis. Ours and others’ research have shown that DNAJB2a (encoded by *DNAJB2* ) is capable of resolving aggregates caused by TDP-43 ( Chen *et al.* , 2016 ), SOD1 ( Novoselov *et al.* , 2013 ; Gess *et al.* , 2014 ), huntingtin ( Labbadia *et al.* , 2012 ) and mutant parkin ( Rose *et al.* , 2011 ). Given DNAJB2’s neuronal enriched expression, it is not surprising that it shows such effectiveness in resolving the protein aggregates associated with neurodegenerative diseases. The question is, is DNAJB2a the sole protein for that role, and is DNAJB2a there to perform such function in the case of neurodegeneration.

ALS is the neurodegenerative disease that most prominently affects motor neurons. It is a heterogeneous disorder where the majority of affected individuals have no family history of ALS. Nevertheless, TDP-43 proteinopathy (i.e. presence of insoluble, hyperphosphorylated, cytosolic TDP-43 aggregates) is found as a common phenotype in affected tissues in ALS with or without mutations in TDP-43. This pathological feature is also seen in ∼60% of FTD which supports the idea that FTD and ALS are on the same disease spectrum. We agree with Frasquet *et al.* ’s conclusion that it would be interesting to study the genetic contribution of DNAJB2a to ALS, but if we do find any, we predict they would be quite different than the ones found in distal hereditary motor neuropathy (dHMN).

The known DNAJB2a mutations in association with dHMN are c.352 + 1G > A ( Blumen *et al.* , 2012 ; Frasquet *et al.* , 2016 ) and c.229 + 1G > A ( Gess *et al.* , 2012 ), both of which are autosomal recessive mutations altering the splicing of DNAJB2 and lead to reduced protein expression. However, in our unpublished study, we did not find the loss of DNAJB2 *per se* is sufficient to trigger TDP-43 aggregation. On the contrary, our data show that HSF1(+) still refolds TDP-43 when DNAJB2a is knocked down (unpublished data). This result suggests that there are some functional redundancy of HSP, and DNAJB2a, although is capable in refolding TDP-43, is not the only HSP for this task. Thereby, a simply loss of DNAJB2 is unlikely to cause TDP-43 proteinopathy that is associated with ALS and FTD. However, as we proposed in our original paper, it does appear that the heat shock response as a whole is compromised in ALS due to a yet to be determined cause. It will be of great interest to investigate the genetic contribution from not only DNAJB2a, but any HSPs and also HSF1.

## Funding

This research was funded principally by a Strategic Grant Award from the Medical Research Council and the Wellcome Trust (grant reference 089701/Z/09/2) to CES with additional support from The Motor Neuron Disease Association, Heaton Ellis Trust, Psychiatry Research Trust and American Amyotrophic Lateral Sclerosis Association.
